# From Ports2Arms: reimagining demand creation for the African context

**DOI:** 10.3389/frhs.2023.1257990

**Published:** 2023-12-19

**Authors:** Perrykent Nkole, Francesca Alice, Alex Stoljar Gold, Lily Yang, Anna W. Matendawafa, Tian Johnson

**Affiliations:** ^1^ The African Alliance, Johannesburg, South Africa; ^2^ Pandemic Emergency Readiness Lab, McGill University, Montreal, QC, Canada

**Keywords:** COVID-19, vaccines, demand creation, inequity, Africa, health rights, accountability, community-led monitoring

## Introduction

The advent of the COVID-19 vaccine was seen as a silver bullet to end the pandemic by many, yet from the outset, African countries faced significant barriers to its effective distribution, access, and uptake. This was compounded by vaccine hoarding and nationalism by global north governments, making procurement difficult. Attempts to “equalise supply access” through global mechanisms like COVAX and bilateral vaccine donations from G7 and EU members (1, 2) (often of expired or close to expired stock) ultimately impeded efficient vaccine distribution and only added to historical mistrust of global north government agendas, with supply continuing to be erratic. As the pandemic progressed, the narrative soon shifted from one of a supply issue to that of distribution and demand, with “demand creation” becoming the central challenge—framed as “Africans don’t want vaccines” (3). Yet, in the context of erratic supply, what can demand creation mean for African communities whose experiences are often left out of vaccine rollout decisions, even when they put their bodies on the line for the research and development of these medical commodities.

Demand creation in the business marketing context refers to the creation of desire or motivation on the part of a consumer to buy a given good or service (4). In the public health context, the product or service is medicine or healthcare, such as a vaccine or medical procedure. In this context, demand creation or generation can be defined as “increas[ing] awareness of, and demand for, health products or services among a particular target audience through social and behavior change communication and social marketing techniques” (5).

What is missing in both the business and public health definitions of demand creation is a nuance that reflects historical legacy and local context. Any successful African vaccination demand creation strategy must be informed by lessons and mistakes from the recent past. Too often awareness is raised for new medical products, for example, new HIV medications in South Africa, resulting in the creation of community-level demand, only to result in a lack of supply when communities ultimately try to access these products. Similar issues occurred with the COVID-19 vaccine as African countries initially faced significant supply shortages, despite the global community touting the importance of the vaccine and the dangers of remaining unvaccinated. This unstable supply created hesitancy and mistrust, ultimately undermining effective demand creation (6). In addition, architects of demand creation strategies must consider any biases they may harbour. As an example, in 2001, Andrew Natsios, then-USAID administrator, claimed that antiretroviral therapy for HIV should not be given to African communities because those “[p]eople do not know what watches and clocks are” and therefore would not be able to take the medications at the right times (7). Not only was this clearly proven false, but evidence demonstrating that Africans are not only more adherent to antiretroviral therapy than North Americans but actively engage with researchers and scientists to ensure that any research or public health intervention considers the local context and community realities so that such processes do not harm communities in the way they are rolled out (8, 9).

In the case of COVID-19, despite millions of vaccines coming in, there is a significant historical legacy to contend with, compounded by overburdened and under-resourced public health systems. Therefore, It is fundamentally immoral to position communities to demand a product before it is provided and before they have received adequate information to make a decision about its use. Communities need accurate and regularly updated information in accessible formats; steady and accessible vaccine supply; and meaningful engagement to better build trust between themselves, government, and vaccine distributors.

While some may argue that to increase vaccination in a time of crisis—like a global pandemic—certain coercive measures are justified (such as mandatory vaccination and associated penalties for unvaccinated workers), community agency in any vaccine rollout is critical for any vaccination program to be successful. The problem with directives regarding preventative measures being exerted in a top-down manner, is that they undermine the trust required for community engagement and can result in decreased demand and potentially less engagement with government, and the research and science community. Indeed, in the face of inconsistent and unreliable COVID-19 vaccine supply, demand decreased, fuelled by mistrust (10). Building agency around demand creation through skills strengthening, access to credible information and ensuring meaningful feedback loops goes hand in hand with community mobilisation; when given the tools, communities will find creative ways to get information out to support vaccination and other preventative measures, but if coercive measures are used, suspicion and resentment may increase, resulting in resistance to vaccination and decreased mobilisation efforts.

## Contextual realities

The community perspective and experience of the COVID-19 pandemic is critical to understanding the role of supply-side inequity in undermining vaccine demand and to chart a more equitable practice for the next pandemic. We need to elevate local voices so we don't replicate the mistakes of the past. A successful demand creation strategy in Africa must come from Africans—grounded in unique national contexts and community realities.

As soon as the COVID-19 vaccine became available, we saw a clear erosion of global solidarity. While African bodies were put on the line as part of fast-tracked clinical trials in South Africa, the subsequent rollout was marked by extreme inequity (11, 12). Individuals in Global North countries received second and third doses well before most Africans received their first vaccine (13). Many African countries were reliant on donations from governments in the Global North, who often provided vaccines based on their own interests and priorities, resulting in a bizarre, disjointed vaccine rollout. Vaccines would arrive with little notice [and often close to expiry (14)], meaning national governments, healthcare workers, and communities had little time to prepare.

In South Africa, disapproval, and mistrust of the government alongside concerns about the effectiveness and potential adverse effects of the vaccine were critical obstacles to the vaccination rollout (15). Rumours contributed to vaccine hesitancy, including those that suggested vaccines were intended by Western countries to “kill Africans”.^1^ Similarly, in Rwanda, some religious communities were said to believe vaccines were an attempt by Westerners to hurt Africans (16). This kind of misinformation, which emerged in the context of doubts about the safety of the AstraZeneca and Johnson and Johnson vaccines, strongly impacted the public perception of COVID-19 vaccines on the continent, casting doubt on the safety of the vaccines in general (17).

Competing priorities also affected community members' desire or willingness to access the vaccine. In the context of climate change and related food insecurity, the costs of travelling to vaccine sites compared to the lost work opportunity costs are significant barriers to getting vaccinated (18). Yet even where vaccines were available, long lines inhibited uptake, or people would get to the end of the queue only to be told that supply had run out. All this led to increasing distrust of the vaccine rollout, and the vaccine itself.

Additionally, in countries like the Democratic Republic of the Congo or on the borders of countries in East Africa, frequent conflict and mass displacement affect people's ability and desire to get vaccinated (19). These access and uptake challenges have been repeatedly compounded by inequitable vaccine supply dynamics globally, leading to confusion around the availability of different vaccine types in which countries, and cynicism and mistrust due to repeated reports of vaccines being delivered close to expiry and having to be destroyed (6). As a result, it is no surprise that this uncertainty and lack of accountability to effective vaccine distribution has significantly limited demand.

## Reimagining demand creation: a rights-based approach

A lack of community ownership was highly evident in vaccine rollout on the continent. Large international bodies with no real contextual experience or understanding made assumptive decisions about local vaccine delivery. For the most part, communities did not have their concerns addressed or have control over when, how, and where vaccines were delivered. In addition, when vaccines were available, many historical and on-the-ground realities resulted in limited uptake and vaccine hesitancy. As a result, effective demand creation must differ from traditional demand creation which often sees communities as passive actors to traditional social marketing techniques. Conversely, a rights-based approach encourages and engages active participation from communities, and recognises that for people to fully realise their right to health, they also must be in the position to claim and realise other rights, such as the right to information and education, and the right to equality under the law and non-discrimination in terms of access. These are expressed through the International Covenant on Economic, Social and Cultural Rights provisions that State parties are obliged to satisfy at minimum essential levels (20):^2^

1. Access to health facilities, goods and services;
2. Access to minimum essential food to ensure freedom from hunger;
3. Access to basic shelter, housing and sanitation;
4. Provision of essential drugs;
5. Equitable distribution of all health facilities, goods and services;
6. Adoption and implementation of a national public health strategy and plan of action based on epidemiological evidence, that addresses the health concerns of the whole population, periodically reviewed in a participatory and transparent process.

In the context of a pandemic, this means that all people at all times have the right to access services and commodities, such as the COVID-19 vaccine, to prevent the exposure to and transmission of a virus like COVID-19, as well as to treatment, care and support services. A community-led approach to demand creation grounded in human rights would first carefully listen to and consider the concerns and needs of community members in its design, seeking to inform them about the benefits of vaccination and provide meaningful, practical support in getting vaccinated, while also acknowledging that every person has a right to make autonomous decisions regarding their health.

This approach differs from the traditional view of demand creation in that, while it encourages vaccination, it does not seek to increase vaccination by *any* means but rather puts agency strengthening and recognition of basic human rights at its centre. To increase demand, communities must regain ownership over the COVID-19 vaccination rollout and general pandemic response, and be supported with accurate information, reliable supply and communication that meets them where they are, as well as allowing for feedback loops to key stakeholders in planning and response. This will better promote trust between communities and vaccine distributors, increasing local motivation and mobilisation efforts for vaccination. Only then can community agency and vaccine confidence be strengthened, and demand creation be truly promoted.

Concretely, what does community ownership of health look like? Our Ports2Arms project seeks to ensure that African communities in all their diversity are meaningfully engaged in monitoring their lived experiences of public health crises, and their documented experiences and advocacy responses inform a shift to local ownership of future prevention, preparedness and response strategies for public health emergencies. This includes local ownership of healthcare delivery in the context of pandemics like COVID-19 (21). It is important to note that this approach does not imply that communities are entirely responsible for vaccination; the state must ensure vaccine availability. It is the role of communities to hold state actors accountable and ensure the vaccine rollout is equitable, just, and accessible to vulnerable populations. This model of community-led monitoring allows for the discussion and integration of various stakeholders and opinions to create effective strategies to improve vaccine uptake.

## Doing better next time

Despite the devastation of the pandemic on the continent and globally, significant good, local practices have emerged to build on to inform future pandemic responses. For instance, in South Africa, local community members rallied to bring vaccines — as well as accurate information — straight to people's homes (22). Pop up vaccination clinics as part of a larger health roadshow have also been a successful initiative. Preparation and local mobilisation must occur *before* the crisis strikes, otherwise it may be too late. Practically, this means learning from COVID-19 — for instance, identifying barriers and enablers to COVID-19 vaccine uptake — so communities can be ready to quickly and efficiently roll out the next vaccine for the next pathogen (21). Focused and locally-tailored interventions are vital to ensure an equitable, rights-based response to the next pandemic or health emergency. What is needed is a constant effort to support communities in taking control over all aspects of their health. When communities lead the response, local agency is strengthened, and the trust that is built creates demand. Only when demand is created within a human rights framework will vaccines be efficiently and equitably transported from “ports to arms”.

## Footnotes

^1^The African Alliance (2023) Semi-structured conversation with communities in Gauteng [transcript]. South Africa.

^2^Internationally, standards for good healthcare commonly use a framework of availability, accessibility, acceptability (including acceptable standards of quality), affordability and accountability.
